# Phylogenetic analysis of the partial sequences of the* env* and* tax* BLV genes reveals the presence of genotypes 1 and 3 in dairy herds of Antioquia, Colombia

**DOI:** 10.1007/s13337-023-00836-9

**Published:** 2023-10-10

**Authors:** Cristina Úsuga-Monroy, F. J. Díaz, Luis Gabriel González-Herrera, José Julián Echeverry-Zuluaga, Albeiro López-Herrera

**Affiliations:** 1 Grupo BIOGEM, Facultad de Ciencias Agrarias, Universidad Nacional de Colombia Sede Medellín, Calle 65 No 59A-110, Medellín, Colombia; 2 Grupo Inmunovirología, Facultad de Medicina, Universidad de Antioquia, Calle 70 No. 52-21, Medellín, Colombia

**Keywords:** Bovine leukemia, Dairy herds, Holstein Friesian, Strain, Virus

## Abstract

Bovine leukemia virus (BLV) is a retrovirus that primarily infects dairy cows. Although few studies have also used the *tax* gene, phylogenetic studies of BLV use mostly the *env* gene. The aim of this work was to establish the circulating genotypes of BLV in specialized dairy cattle from Antioquia, Colombia. Twenty blood samples from Holstein Friesian cows were collected, and their DNA was isolated. A PCR was performed for a partial region of the *env* and *tax* genes. A phylogenetic analysis was carried out using the maximum likelihood and Bayesian methods for both genes. Nineteen sequences were identified as genotype 1 by *env* and *tax* genes. Only one sequence was clustered with genotype 3 and had the highest proportion of different nucleotide sites compared to other strains. Four amino acid substitutions in the 134 amino acid residue fragment of the Env protein were identified in the Colombian sequences, and three new amino acid substitutions were reported in the 296 amino acid residue fragment of the Tax protein. R43K (Z finger), A185T (Activation domain), and L105F changes were identified in the genotype 3 sample. This genotype has been reported in the United States, Japan, Korea, and Mexico, but so far, not in Colombia. The country has a high rate of imported live animals, semen, and embryos, especially from the United States. Although it is necessary to evaluate samples from other regions of the country, the current results indicate the presence of two BLV genotypes in specialized dairy herds.

## Introduction

The etiologic agent of enzootic bovine leukemia (EBL) is the bovine leukemia virus (BLV) that belongs to the *Deltaretrovirus* genus and the *Retroviridae* family. BLV infects bovine B cells and compromises the humoral immune response. Among 30 to 70% of infected cows have increased blood lymphocyte count; between 0.1 and 10% of the cows with more than three years of infection develop the tumor phase or lymphosarcomas [19, 32]. The BLV genome has a size of 8714 base pairs and has a *pX* region flanked by two identical long terminal repeats (LTRs), in addition to the *gag*, *pro*, *pol*, and *env* gene regions [1, 28]. The *gag* gene codes for p15, p24, and p12 proteins. The p15 or matrix protein, has 109 amino acids; the p12 or nucleocapsid protein, has 69 amino acids and is strongly bound to the viral RNA; and the virus capsid consists mainly of the p24 protein. The *pol* gene codes for integrase and reverse transcriptase [14, 28]. The *env* gene codes for the gp51 surface glycoprotein and the gp30 transmembrane protein; both proteins are glycosylated polypeptides and are associated through disulfide bridges forming a stable complex. The gp51 protein plays an essential role in the life cycle of the virus since it requires to enter the cell, induces the mass production of neutralizing antibodies in infected animals, and is essential for the infectivity of the virus [4, 14]. Moreover, the N-terminal of the gp51 protein has three conformational epitopes (F, G, and H) associated with viral infectivity and syncytia formation [4].

The pX region of the BLV genome contains the genes *tax*, *rex*, *G4,* and *R3* related to regulatory viral transcription functions. The *tax* gene is a transcriptional transactivator that increases transcription factor binding to DNA and upregulates the synthesis of viral proteins. The tax protein acts on the enhancer motif Tax-response element (TxRE) located in the 5′LTR’s U3 region of the BLV genome to stimulate viral transcription [2, 43]. The tax protein has oncogenic potential, inhibits DNA repair, increases DNA damage by the accumulation of mutations [14], contributes to the induction of lymphomas in mouse fibroblasts through cooperation with the oncoprotein Ha-Ras [33, 37], modulates the expression of genes related to cell growth, affects the proliferation of B lymphocytes, cell survival and the independent growth of cytokines in B cells [35].

A phylogenetic tree is an ideal approach for reconstructing the evolutionary history of any organism on earth and understanding adaptive evolution at the molecular level. Sometimes, the mutational changes of DNA sequences determine changes in protein synthesis that promote genetic variants. The *env* gene is the most used for phylogenetic studies of BLV, and its characterization has resulted in a classification of BLV in 12 genotypes [34]. According to Nishikaku et al. [24], after the first appearance of BLV in Asia, the progenitor of lineage I reached Europe in 1824, and from there, it reached South America in 1850. From South America, it passed to the United States and Mexico in 1942 [24]. BLV differences at the nucleotide level have been related to geographic location; therefore, the geographic component plays an essential role in BLV distribution worldwide. Nucleotide differences in many *env* sites are related to the geographical location of the strain. Genotypes 7 and 8 have been registered only in Europe, genotype 9 only in Bolivia [29], genotype 10 only in Thailand [18], and the new genotype 12 in Kazakhstan [34]. However, genotypes 1 and 4 are present on several continents. BLV is widely distributed in the Americas. Genotypes 1 and 5 are found in the United States, genotypes 1 and 5 in Costa Rica [42], genotypes 1, 2, and 6 in Brazil [6], genotype 7 in Chile [12], genotype 1 in Uruguay [23], and genotype 9 in Bolivia [29]. On the other hand, reports indicate that the *tax* gene is used to confirm the presence of BLV infection [10, 27] and is also used in BLV phylogenetic studies [8, 9]. The *tax* gene is more divergent than the pol gene, therefore its sequence could provide significant information for virus characterization [9]. Accordingly, the aim of this work was to establish the circulating genotypes of BLV in the specialized dairy cattle of Antioquia, Colombia using partial sequences of the *env* and *tax* genes through two f phylogenetic inference methods.

## Materials and methods

### Samples

Blood samples were collected from 20 Holstein Friesian cows between the first and fifth lactation period and from 3 to 7 years of age. Sampling was carried out from February to June 2017. Cows naturally infected with BLV were previously diagnosed by ELISA (SVANOVIR® BLV gp51-Ab) for the gp51 protein and by PCR [40] for the envelope gene. None of the selected cattle showed visible symptoms associated with BLV and were milking. These animals came from 10 municipalities of Antioquia divided into three subregions: North (Belmira, Entrerios, San Pedro de los Milagros, Don Matias, and Santa Rosa de Osos), Valle de Aburrá (Bello and Medellín), and East (La Unión, Rionegro, and El Retiro). Two municipality-level samples were selected for sequencing.

### DNA isolation

One tube (4 mL) of blood was collected in heparinized syringes by coccygeal venipuncture. Samples were homogenized by inversion and transferred to 15 mL tubes. The *buffy coat* was isolated by centrifugation for 4 min at 3000 rpm and 4 °C. The salting-out technique obtained DNA from the *buffy coat* [20]. DNA was resuspended in TE buffer (1 M Tris HCl, 0.5 M EDTA, pH 8.0) and stored at 4 °C until analysis. The quality and quantity of the DNA were evaluated on a spectrophotometer (NanoDrop2000) and agarose gel (1%).

### PCR amplification for the env gene

A nested PCR was performed for all samples. A region of the viral *env* gene was amplified to obtain a 444 bp fragment using the previously reported oligonucleotides [5]. The reaction was carried out according to the protocol previously described [40].

### PCR amplification for the tax gene

A region of the viral *tax* gene was amplified to obtain a 926 bp fragment using the oligonucleotides of this study, tax-FW (5′-GGCCCCACTCTCTACATGC-3′) and tax-RV (5′-CGGGAGAGCCATTCATTTT-3′). PCR amplification was performed in a final volume of 25 μL with 150 ng DNA, 3.0 μL (10 mM) of each oligonucleotide, 0.4 mM dNTPs, 1X buffer PCR (ThermoScientific®), 3 mM MgCl_2_, and 1U Taq Polymerase DNA. The reaction was performed in a T3 thermocycler (Biometra®) with the following protocol: initial denaturation at 94 °C for 5 min, followed by 40 cycles of 98 °C for 30 s, 56 °C for 30 s, and 72 °C for 3 min; the final extension was run at 72 °C for 5 min. The PCR product was analyzed by electrophoresis on 2% agarose gel stained with EZ-VISION (Amresco®) in a Gel Doc (BioRad, California-USA).

### Sequencing

The PCR products obtained from the samples were sent for sequencing in a commercial facility (Macrogen Inc. Korea). The PCR products were sent in 1.5 mL tubes at 100 ng/μL concentrations in a final volume of 30 μL. Sequencing was done in both senses. The same commercial facility purified the products.

### Phylogenetic analysis of env nucleotide sequences

Nucleotide sequences were aligned with 52 partial sequences of the *env* gene deposited in GenBank representative of the 12 BLV genotypes. These sequences were from different geographic regions, including several South American countries. Sequences were manually aligned in the software MEGA v11. The Hasegawa-Kishino-Yano with Gamma distribution (HKY + G) was selected as the best substitution model based on the Akaike Information Criterion (AIC). Phylogenetic analyses were performed by two methods: (1) a Maximum Likelihood analysis was performed using MEGA v11 branch support determined with 1000 bootstraps, and (2) a second phylogenetic tree was built utilizing the Bayesian method with the MrBayes V3.2.7 program with the same substitution model. The number of generations was determined until the likelihood of the cold chain (LnL) was stationary. The final tree was edited in FigTree V1.4.0. Mean nucleotide distances within (intra-genotype) and between (inter-genotype) BLV genotypes were estimated and compared using the *p-distance* model, and the nucleotide substitution rate matrix was estimated using the maximum likelihood method in MEGA v11.

### Phylogenetic analysis of tax nucleotide sequences

For the phylogenetic analysis of the *tax* region, sequences of the 19 BLV strains of this study were aligned with 55 sequences obtained from GenBank. For this analysis, complete genome sequences of BLV and the *pX* region were included. Sequences were manually aligned in the software MEGA v11. Phylogenetic analysis was performed using the same two methods used for *env*. First, a Maximum Likelihood analysis using MEGA v11 was performed using the General Time Reversible with Gamma distribution (GTR + G), selected as the best substitution model based on AIC, bootstrap values were determined with 1000 repetitions. A second phylogenetic tree was built by Bayesian methods using MrBayes V3.2.7 and the evolution model used was General Time Reversible with Gamma distribution (GTR + G) distribution with the same substitution model. The number of generations was determined until the likelihood of the cold chain (LnL) was stationary. Mean nucleotide distances within (intra-genotype) and among (inter-genotype) and the nucleotide substitution rate matrix were estimated in the same way as for the *env* gene.

### Amino acid changes in BLV envelope protein (Env) and regulatory protein (Tax)

Alignments of the partial amino acid sequences of Env and Tax proteins from the twenty strains were obtained using MEGA v11 tools. The BLV cell line FLK (GenBank accession EF600696), BLV strains Arg41 (GenBank accession FJ914764), and the complete BLV genome (GenBank accession AF033818) were used as reference sequences for the analysis of amino acid changes.

## Results

### Phylogenetic analysis of env nucleotide sequences

**Fig. 1:**
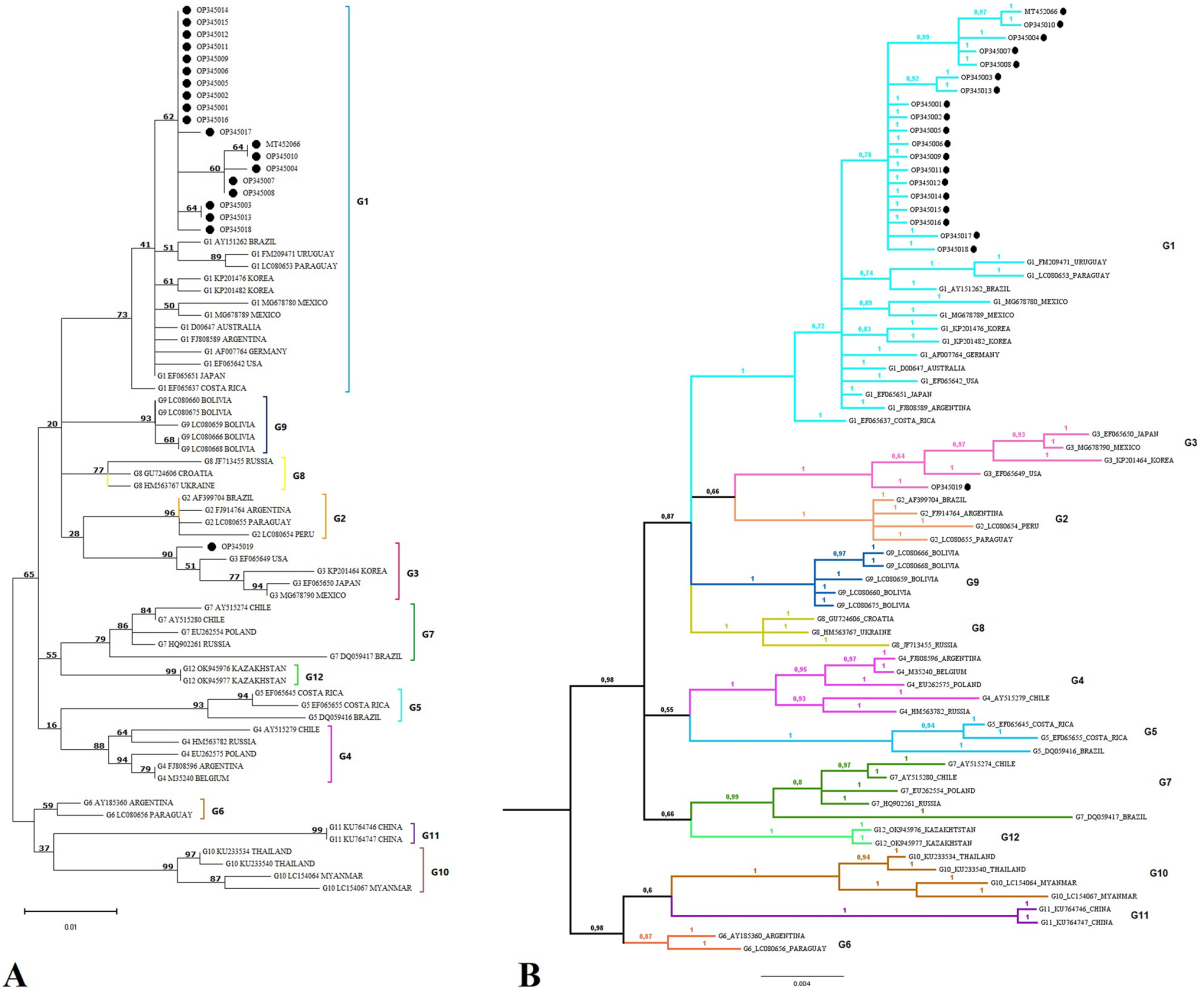
Molecular phylogenetic analysis of the BLV *env* gene (444 nucleotides) through Maximum Likelihood and Bayesian inference **A**. Maximum Likelihood: The evolutionary history was inferred using the Hasegawa-Kishino-Yano substitution model. A discrete Gamma distribution was used to model evolutionary rate differences among sites. Branch support values came from 1000 bootstrap replicates. The tree is drawn to scale, with branch lengths measured in the number of substitutions per site. Bar length indicates 0.01 substitutions per site. Twenty partial *env* sequences from Colombian municipalities are indicated with (●). Genotypes (G) are indicated on the right side. There were a total of 444 positions and 72 sequences in the final dataset. Evolutionary analyses were conducted in MEGA v11. **B** Bayesian phylogenetic tree of the BLV *env* gene sequences inferred with the Hasegawa-Kishino-Yano model with Gamma distribution (HKY + G). The consensus tree was obtained after discarding (burnin) the initial 25% of the generations. The numbers at the nodes show posterior probabilities, and the bar length indicates 0.003 substitutions per site. Partial *env* gene sequences from Colombian municipalities are indicated with (●). Genotypes (G) names are shown around the figure. A total of 444 positions and 72 sequences were included in the final dataset. Evolutionary analysis was conducted in MrBayes V3.2.7

The partial sequences of the *env* gene were analyzed from strains obtained from Holstein Friesian cows from the Department of Antioquia. A PCR product of 444 bp was obtained for the *env* gene, and the samples were identified as follows: OP345001, OP345002, OP345003, OP345004, OP345005, OP345006, OP345007, OP345008, OP345009, OP345010, OP345011, OP345012, OP345013, OP345014, OP345015, OP345016, OP345017, OP345018, OP345019, and MT452066. A Maximum Likelihood phylogenetic tree of the *env* gene (Fig. 1A) sequences of 19 Colombian samples formed a monophyletic group with < 70% bootstrap support inside genotype 1. Sequences from Brazil, Uruguay, Argentina, Mexico, Costa Rica, the United States, Korea, and Japan were also in genotype 1. The complete genotype 1 clade had a 73% bootstrap support. The OP328419 Belmira sample was the only one that grouped with the sequences EF065649 (United States), KP201464 (Korea), EF065650 (Japan), and MG678790 (Mexico) in genotype 3 with 90% bootstrap support. Figure 1B shows the resulting Bayesian phylogenetic tree. As in the Maximum Likelihood tree, 19 Colombian sequences were grouped with sequences from Brazil, Uruguay, Paraguay, Mexico, Argentina, the United States, and Korea within genotype 1. On the contrary, the OP328419 Belmira sample grouped with sequences from the EF065649 (United States), KP201464 (Korea), EF065650 (Japan), and MG678790 (Mexico) in genotype 3 with high posterior probabilities (prob = 1).

As shown in Table 1, the mean intra-genotype distances between genotypes 4, 5 and 10 are not different (0.014). Genotypes 5 and 10 (0.056) and 3 and 10 (0.051) are larger than genotypes 1 and 10, and 7 and 10 (0.047). On the other hand, genotypes 8 and 10 have the smallest mean inter-genotype distance (0.022).

**Table 1:** Mean intra-genotype and inter-genotype genetic distances in the *env* gene (444 bp) of BLV genotypes. Distances were estimated with the *p-distance* substitution model

	G.1	G.2	G.3	G.4	G.5	G.6	G.7	G.8	G.9	G.10	G.11	G.12
G.1	0.008*											
G.2	0.031	0.006*										
G.3	0.033	0.030	0.012*									
G.4	0.034	0.028	0.038	0.014*								
G.5	0.036	0.037	0.045	0.037	0.014*							
G.6	0.029	0.030	0.032	0.027	0.038	0.008*						
G.7	0.036	0.032	0.040	0.034	0.040	0.030	0.017*					
G.8	0.025	0.026	0.029	0.028	0.040	0.023	0.031	0.010*				
G.9	0.027	0.028	0.030	0.028	0.041	0.026	0.032	0.022	0.003*			
G.10	0.047	0.048	0.051	0.044	0.056	0.030	0.047	0.040	0.044	0.014*		
G.11	0.054	0.050	0.057	0.048	0.057	0.035	0.050	0.048	0.047	0.051	0.000*	
G.12	0.033	0.034	0.037	0.031	0.039	0.027	0.028	0.028	0.030	0.045	0.047	0.000*

Bold values: *Mean intra-genotype nucleotide distances

The BLV sequences OP345005 (El Retiro), OP345006 (El Retiro), OP345015 (San Pedro), and OP345016 (San Pedro) are identical using the *p-distance* model, although they are from distant municipalities geographically separated by a valley. OP345019 (Belmira) has a higher proportion of nucleotide sites different from other samples and is farther from the OP345010 (La Unión) and OP345004 (Donmatias) sequences (bold cells) (Table 2).

**Table 2:** Estimates of evolutionary divergence between twenty BLV strains from different municipalities of Antioquia, Colombia. Distances were estimated with the *p-distance* substitution model

		1	2	3	4	5	6	7	8	9	10	11	12	13	14	15	16	17	18	19	20
1	BELLO_006 OP345001																				
2	BELLO_364 MT452066	0.008																			
3	BELMIRA_108 OP345019	0.023	0.030																		
4	BELMIRA_9911 OP345002	0.000	0.008	**0.023**																	
5	DONMATIAS_026 OP345003	0.003	0.010	**0.025**	0.003																
6	DONMATIAS_053 OP345004	0.008	0.005	**0.030**	0.008	0.010															
7	ELRETIRO_6177 OP345005	0.000	0.008	**0.023**	0.000	0.003	0.008														
8	ELRETIRO_P16 OP345006	0.000	0.008	**0.023**	0.000	0.003	0.008	0.000													
9	ENTRERRIOS_126 OP345007	0.005	0.003	**0.028**	0.005	0.008	0.003	0.005	0.005												
10	ENTRERRIOS_177 OP345008	0.005	0.003	**0.028**	0.005	0.008	0.003	0.005	0.005	0.000											
11	LAUNION_5682 OP345009	0.000	0.008	**0.023**	0.000	0.003	0.008	0.000	0.000	0.005	0.005										
12	LAUNION_5719 OP345010	0.008	0.000	**0.030**	0.008	0.010	0.005	0.008	0.008	0.003	0.003	0.008									
13	MEDELLIN_421 OP345011	0.000	0.008	**0.023**	0.000	0.003	0.008	0.000	0.000	0.005	0.005	0.000	0.008								
14	MEDELLIN_P14 OP345012	0.000	0.008	**0.023**	0.000	0.003	0.008	0.000	0.000	0.005	0.005	0.000	0.008	0.000							
15	RIONEGRO_375 OP345013	0.003	0.010	**0.025**	0.003	0.000	0.010	0.003	0.003	0.008	0.008	0.003	0.010	0.003	0.003						
16	RIONEGRO_P17 OP345014	0.000	0.008	**0.023**	0.000	0.003	0.008	0.000	0.000	0.005	0.005	0.000	0.008	0.000	0.000	0.003					
17	SANPEDRO_118 OP345016	0.000	0.008	**0.023**	0.000	0.003	0.008	0.000	0.000	0.005	0.005	0.000	0.008	0.000	0.000	0.003	0.000				
18	SANPEDRO_137 OP345015	0.000	0.008	**0.023**	0.000	0.003	0.008	0.000	0.000	0.005	0.005	0.000	0.008	0.000	0.000	0.003	0.000	0.000			
19	SANTAROSA_1750 OP345017	0.003	0.005	**0.025**	0.003	0.005	0.010	0.003	0.003	0.008	0.008	0.003	0.005	0.003	0.003	0.005	0.003	0.003	0.003		
20	SANTAROSA_1758 OP345018	0.003	0.010	**0.025**	0.003	0.005	0.010	0.003	0.003	0.008	0.008	0.003	0.010	0.003	0.003	0.005	0.003	0.003	0.003	0.005	

Bold values indicate the evolutionary divergence between OP345002 and other BLV strains

Substitution pattern and rates were estimated under the General Time Reversible model (+G); the nucleotide frequencies are A = 23.42%, T/U = 26.97%, C = 29.25%, and G = 20.36% for the *env* gene fragment (Table 3).

**Table 3:** Maximum Likelihood estimates of the substitution matrix (*env* gene)

	A	T/U	C	G
A	–	*1.52*	*3.46*	**18.38**
T/U	*1.32*	–	**23.72**	*1.09*
C	*2.77*	**21.88**	–	*1.34*
G	**21.15**	*1.45*	*1.92*	–

Rates of different transitional substitutions are shown in bold, and those of transversional substitutions are observed in italics

### Phylogenetic analysis of tax nucleotide sequences

**Fig. 2:**
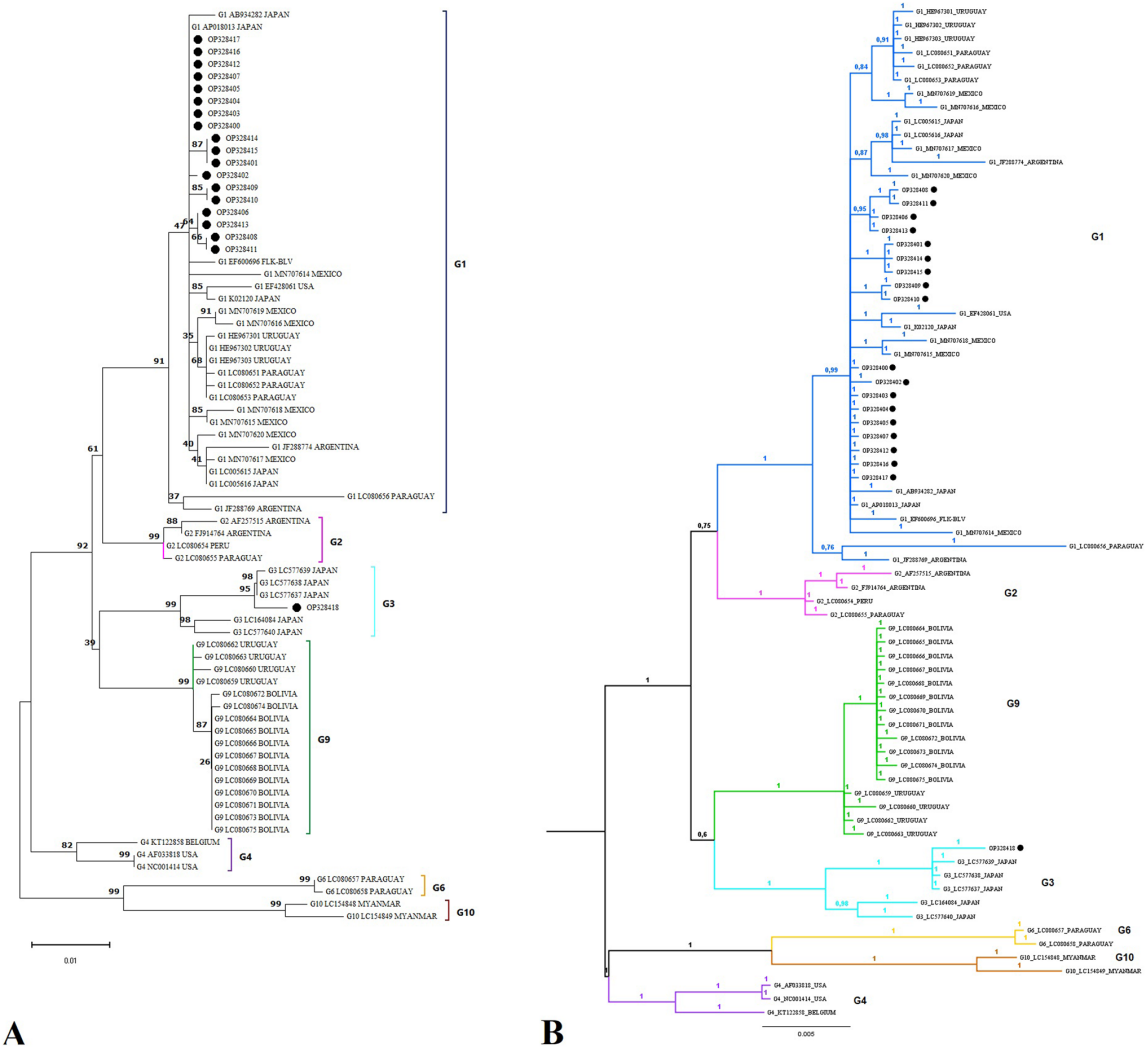
Phylogenetic tree of the BLV *tax* gene by Maximum Likelihood and Bayesian inference. **A** Maximum Likelihood method: The evolutionary history was inferred using the General Time Reversible substitution model with a discrete Gamma distribution of rate differences among sites (GTR + G). Bootstrap values (1000 replicates) are shown close to the nodes. The tree is drawn to scale, with branch lengths measured as the number of substitutions per site (Bar: 0.01 substitutions per site). The analysis involved 55 partial *tax* gene sequences from the GenBank database. Nineteen *tax* gene sequences from Colombian municipalities are indicated with (●). Genotypes (G) are indicated by the numbers to the right of the clades. There was a total of 926 positions in the final dataset. Evolutionary analyses were conducted in MEGA v11. **B** Bayesian phylogenetic tree using the General Time Reversible model with Gamma distribution (GTR + G). The numbers at the nodes are posterior probabilities, and the bar length indicates 0.005 substitutions per site. The analysis involved 74 partial *tax* gene sequences. Partial *tax* gene sequences from Colombian municipalities are indicated with (●). Numbers around the figure indicate genotypes. There was a total of 926 positions in the final dataset. Evolutionary analyses were conducted in the MrBayes V3.2.7 program

Figure 2A presents the Maximum Likelihood phylogenetic tree based on 926 bp of *tax* gene sequences of BLV strains. The sample RIONEGRO_375 was not successful in sequencing. Eighteen Colombian sequences were grouped in genotype 1 with 91% bootstrap support, along with sequences from Japan (AB934282, AP018013), Mexico (MN707614), Uruguay (HE967301), and Argentina (JF288774). The OP345019 (Belmira) sequence from the *tax* gene grouped in genotype 3 with sequences from Japan (LC577639, LC577638, and LC577637); the bootstrap support of this branch was 95%. The topology of the Bayesian phylogenetic tree of the *tax* gene was similar to the Maximum Likelihood tree. Eighteen Colombian sequences were grouped in genotype 1 with high posterior probabilities (prob = 1) and with sequences from Uruguay, Paraguay, Mexico, Argentina, USA, and Japan. The sequences of strains from Bolivia and Paraguay are found within genotype 9. On the other hand, the OP345019 (Belmira) sequence was registered in the same clade as genotype 3 with sequences from Japan (the same sequences used in the Maximum Likelihood tree).

Table 4 shows the mean intra-genotype and inter-genotype nucleotide distance in the *tax* gene (926 bp). The mean intra-genotype distances between genotypes 4 and 10 are not different (0.014). This distance was similar to the analysis of the *env* gene. Genotypes 1 and 6, 3 and 6, and 3 and 10 have the biggest mean inter-genotype distance (0.056). Genotypes 1 and 2 and 2 and 9 have the smallest mean inter-genotype distance (0.022).

**Table 4:** Mean intra-genotype and inter-genotype genetic distances in the *tax* gene (926 bp). Distances were estimated with the *p-distance* substitution model

	G.1	G.2	G.3	G.4	G.6	G.9	G.10
G.1	0.006*						
G.2	0.023	0.004*					
G.3	0.032	0.030	0.011*				
G.4	0.034	0.029	0.037	0.010*			
G.6	0.056	0.052	0.056	0.044	0.001*		
G.9	0.028	0.023	0.031	0.029	0.055	0.002*	
G.10	0.052	0.049	0.056	0.043	0.044	0.051	0.010*

Bold values: *Mean intra-genotype nucleotide distances

BLV sequences from Santa Rosa and El Retiro municipalities are identical using the *p-distance* model, although they are geographically remote municipalities. OP328418 Belmira has a higher proportion of nucleotide sites than other samples and is farther from the San Pedro, La Unión, Entrerríos, and Medellín sequences (bold cells) (Table 5).

**Table 5:** Estimates of evolutionary divergence between nineteen BLV strains from different municipalities of Antioquia, Colombia. Distances were estimated with the *p-distance* substitution model

		1	2	3	4	5	6	7	8	9	10	11	12	13	14	15	16	17	18	19
1	BELLO_006 OP328400																			
2	BELLO_364 OP328401	0.002																		
3	BELMIRA_108 OP328418	0.028	0.030																	
4	BELMIRA_9911 OP328402	0.001	0.003	**0.029**																
5	DONMATIAS_026 OP328403	0.000	0.002	**0.028**	0.001															
6	DONMATIAS_053 OP328404	0.000	0.002	**0.028**	0.001	0.000														
7	ELRETIRO_6177 OP328405	0.000	0.002	**0.028**	0.001	0.000	0.000													
8	ELRETIRO_P16 OP328406	0.001	0.003	**0.029**	0.002	0.001	0.001	0.001												
9	ENTRERRIOS_126 OP328407	0.000	0.002	**0.028**	0.001	0.000	0.000	0.000	0.001											
10	ENTRERRIOS_177 OP328408	0.002	0.005	**0.030**	0.003	0.002	0.002	0.002	0.001	0.002										
11	LAUNION_5682 OP328409	0.002	0.005	**0.030**	0.003	0.002	0.002	0.002	0.003	0.002	0.005									
12	LAUNION_5719 OP328410	0.002	0.005	**0.030**	0.003	0.002	0.002	0.002	0.003	0.002	0.005	0.000								
13	MEDELLIN_421 OP328411	0.002	0.005	**0.030**	0.003	0.002	0.002	0.002	0.001	0.002	0.000	0.005	0.005							
14	MEDELLIN_P14 OP328412	0.000	0.002	**0.028**	0.001	0.000	0.000	0.000	0.001	0.000	0.002	0.002	0.002	0.002						
15	RIONEGROP17 OP328413	0.001	0.003	**0.029**	0.002	0.001	0.001	0.001	0.000	0.001	0.001	0.003	0.001	0.001	0.000					
16	SANPEDRO_118 OP328414	0.002	0.000	**0.030**	0.003	0.002	0.002	0.002	0.003	0.002	0.005	0.005	0.005	0.005	0.002	0.003	0.003			
17	SANPEDRO_137 OP328415	0.002	0.000	**0.030**	0.003	0.002	0.002	0.002	0.003	0.002	0.005	0.005	0.005	0.005	0.002	0.003	0.003	0.000		
18	SANTAROSA_1750 OP328416	0.000	0.002	**0.028**	0.001	0.000	0.000	0.000	0.001	0.000	0.002	0.002	0.002	0.002	0.000	0.001	0.001	0.002	0.002	
19	SANTAROSA_1758 OP328417	0.000	0.002	**0.028**	0.001	0.000	0.000	0.000	0.001	0.000	0.002	0.002	0.002	0.002	0.000	0.001	0.001	0.002	0.002	0.000

Bold values indicate the evolutionary divergence between OP345002 and other BLV strains

Substitution patterns and rates were estimated under the General Time Reversible model (+G). The nucleotide frequencies are A = 18.58%, T/U = 24.34%, C = 37.44%, and G = 19.64% for the *tax* gene fragment (Table 6).

**Table 6:** Maximum Likelihood estimate of the substitution matrix (*tax* gene)

	A	T/U	C	G
A	–	*2.10*	*6.17*	**23.60**
T/U	*1.60*	–	**22.20**	*0.22*
C	*3.06*	**14.43**	–	*1.38*
G	**22.32**	*0.28*	*2.63*	–

Rates of different transitional substitutions are shown in bold, and those of transversional substitutioins are shows in italics

### Amino acid sequences of the BLV envelope protein (Env)

**Fig. 3:**
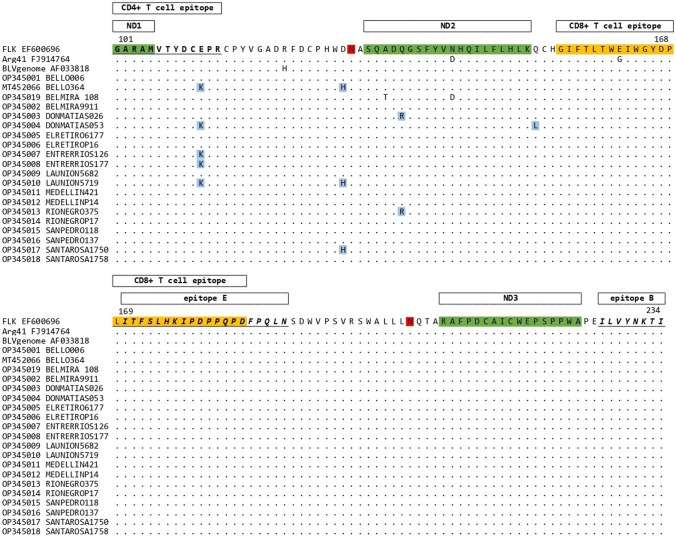
Alignment of the partial amino acid sequences of the envelope protein (Env) of twenty strains from Colombia. The homologous residues of the FLK (EF600696), Arg41 (FJ914764), and the complete BLV genome (AF033818) reference strains were also included for comparison purposes. Amino acids involved in neutralization domains are shown in green. The CD4 + T epitopes are in bold and underlined. The CD8 + T epitopes are shown in orange. Linear epitopes, B and E, are shown in italics and underlined. N-glycosylation sites are in red. Newly found substitutions are marked with blue. Epitopes and glycosylation sites were obtained from previous reports [22, 29, 31]

Figure 3 presents the amino acid alignment of the Env protein for the 20 strains obtained from Holstein Friesian cows naturally infected with BLV. The envelope protein of 134 amino acids was obtained from the amino acid positions 101 to 234. Four different amino acid substitutions were found in the Colombian sequences concerning reference sequences and previously reported changes. E111K was found in five strains (MT452066, OP345004, OP345007, OP345008, and OP345010), D128H in three (MT452066, OP345010, and OP345017), Q135R in two (OP345003 and OP345013), and Q151L only in OP345004. E111K is in the CD4 + T cell epitope, whereas Q135R is in the neutralization domain ND2.

### Amino acid sequences of the BLV regulatory protein (Tax)

**Fig. 4:**
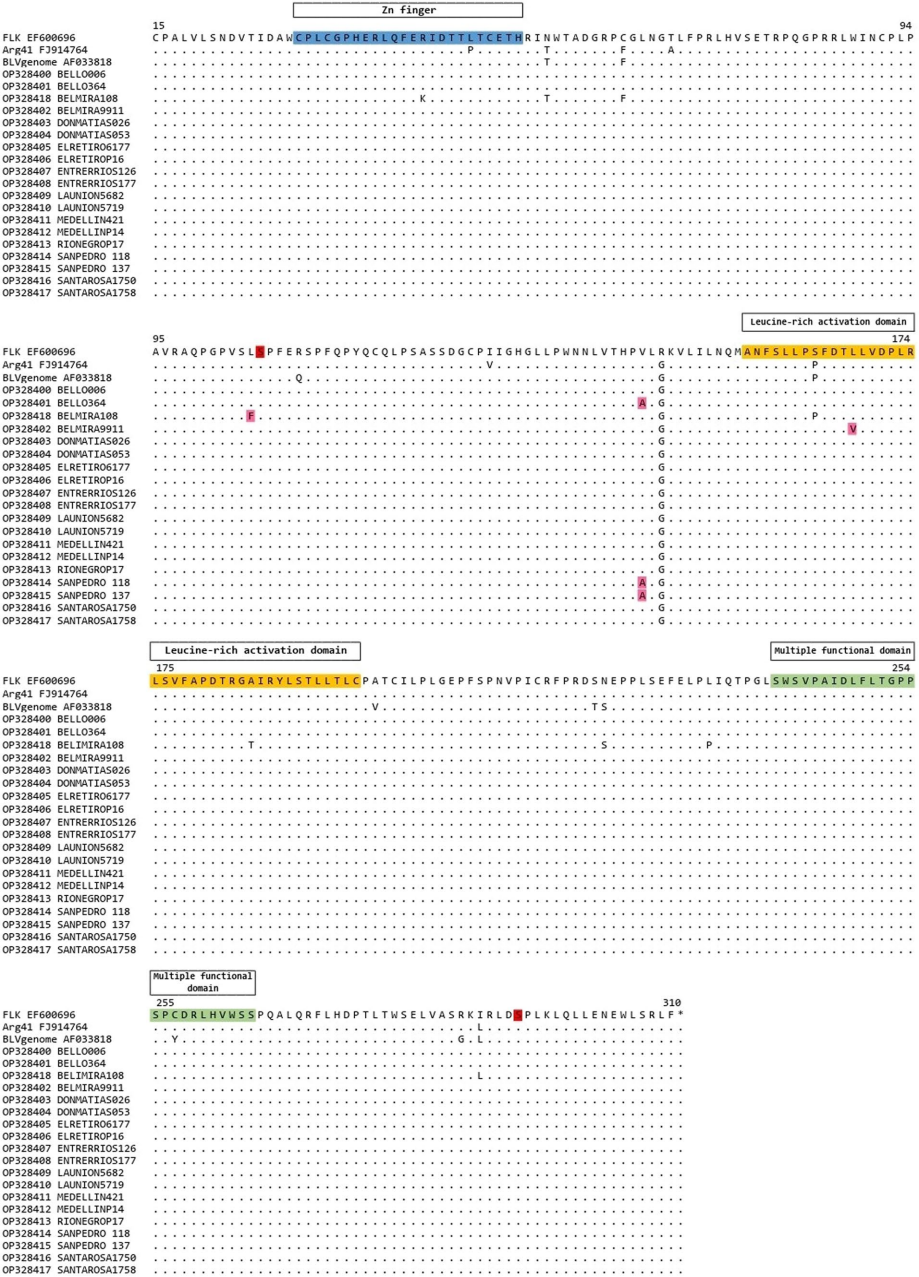
Alignment of the partial amino acid sequences of the regulatory Tax protein of the 19 Colombian BLV strains obtained in this study. The homologous sequences of FLK (EF600696), Arg41 (FJ914764), and the complete BLV genome (AF033818) reference strains were included. The location of a putative zinc finger domain (blue), a Leucine-rich activation domain (yellow), a multiple functional domain that influences the transactivation activity of the Tax protein (green), and phosphorylation sites (red) are highlighted. Newly found substitutions are marked with pink. Domains and multiple functional domain sites were obtained from previous reports [22, 29, 31]

Figure 4 shows the amino acid alignment of the partial sequence of the Tax protein for 19 BLV strains. The 296 amino acid residues (positions 15 to 310 in the protein) were deduced from the nucleotide sequence of the *tax* gene obtained in this study. Three new amino acid substitutions were found in the strains relative to the reference sequences and previously reported amino acid changes. R43K and A185T were found in OP328418 (Belmira strain), previously reported by Ohnuki et al. [30]. However, a new change in L105F was found in this same strain. The V146A amino acid change (without a previous report) was registered in three strains (OP328401, OP328414, and OP328415). The A185T and L168V changes (without a previous report) are recorded in the leucine-rich activation domain.

## Discussion

The BLV genotypes were initially established from the differences found in the restriction site analysis, and the BLV strains were classified into three genotypes according to the restriction patterns of Beier et al. [5]. Currently, BLV is classified into twelve genotypes described through the partial sequence of the *env* gene. The topology used in the phylogenetic analyzes of the *env* gene with high values of branch support (> 70% in bootstrap and 1.0 in posterior probabilities) by the maximum likelihood and Bayesian methods agrees with the topology published in other papers for the 12 current genotypes [18, 28, 42]. Through these analyses, genotypes 1 and 3 were identified in the samples of the municipalities of Antioquia. Genotype 1 has previously been identified in South America in Brazil, Uruguay, Argentina, and Mexico (in Holstein Friesian Cows) [6, 21, 23, 30]. Studies have found a correlation between the genotype and the geographical origin of the sample [24]. Genotype 1 is distributed worldwide [15], but some genotypes have been found in specific regions. Genotypes 6 and 9 are found in South America only [29]; Genotype 8 solely in Europe [31]; Genotype 10 only in Southeast Asia [18]; and recently, a new genotype was discovered in Kazakhstan [34]. The origin of BLV is located in Asia, and its distribution throughout the world is due to its ability to infect cattle (related to primary production and food security). The introduction of cows in South America and later to the United States (1950) and the crosses with local populations positively influenced the rapid spread of the virus [24]. In addition, geographic isolation has also contributed to the diversification of the virus [18, 29, 34].

The current research identified one sample of OP328418 (by the *env* gene) and OP345019 (by the *tax* gene) from Belmira as genotype 3. This genotype has previously only been reported in Korea [17], the United States, and Japan [42] and was recently identified in Mexico [7]. The Belmira strain is the first genotype 3 reported in South America, and its entry into the country may also be related to the import of live animals, semen, and embryos, especially from the United States. In 2017, 108 breeding cattle, 118 embryos, and 331,057 semen straws were imported to Colombia. The United States has been one of the primary sources of genetic material (semen or embryo samples) for genetic breeding programs of cattle herds in Colombia. The BLV genome has been found in fresh and frozen semen samples [3, 11]. Semen, like other fluids such as blood, are sources for the horizontal transmission of BLV [11], and its infectivity potential has been demonstrated in the sheep model [16].

The transcript of the *env* gene is a 5.1 kb RNA; the resulting products are the surface protein (SU) or gp51 and the transmembrane protein (TM) or gp30. Both proteins are glycosylated polypeptides and are associated through disulfide bridges forming a stable complex [14]. The gp51 protein is responsible for binding the virus to the host cell. This interaction allows the refolding of the gp30 protein, which activates its potential to fuse with the plasma membrane of the cell through a fusion peptide. The gp51 protein induces the production of antibodies in infected animals and is essential for the infectivity of the virus [14]. On the other hand, the gp51 protein has three conformational epitopes (F, G, and H) in the N-terminal region, which play an important role in viral infectivity and syncytia formation [4]. The Env protein has four important regions: CD4 + epitope, zinc-binding peptide, CD8 + T cell epitope, and E epitope. This study found four new amino acid substitutions: E111K substitution was the most frequent among the Colombian strains, followed by D128H, Q135R, and Q151L. The change of amino acid E111K was registered in the CD4 + T cell epitope, whereas the change of amino acid Q135R was recorded in the neutralization domain ND2. CD4 + T cell epitope has been related to the protection against infections and cancer [39]. The substitution of glutamine (polar amino acid uncharged) by arginine (an amino acid with a positive charge) can change the structure or function of this domain. Substitutions between residues 137 and 156 affect the fusion of the gp51 protein and in vivo infectivity [13]. It also decreases the immunoreactivity of the ND2 epitope [41]. Only one strain showed a change in this region: the change of glutamine (polar amino acid uncharged) by leucine (hydrophobic amino acid) in position 151. However, the effect of this substitution on the protein structure is not known. CD8 + T cell epitope, E epitope, and ND3 epitope were conserved in all the strains from Colombia.

BLV clusters have generally been elaborated through phylogenetic analysis of a 444 bp fragment of the envelope gene (*env*); however, other studies have also used the viral *tax* gene [8, 9]. The phylogenetic analyses carried out in the current study through the Bayesian and Maximum Likelihood of the *env* and *tax* genes of Colombian strains are consistent in the genotype 1 and genotype 3 clustering. The mean inter-genotypic nucleotide distances in the *env* and *tax* genes are similar. This suggests that *tax* is a potential gene for phylogenetic studies. The *tax* gene is a transcriptional transactivator that increases transcription factors binding to DNA and improves the synthesis of viral proteins. It is located in the *pX* region between the env and the 3′LTR regions [2]. The tax protein targets T cells through epitopes 110–130/131–150 and B cells through epitopes 261–280. It modulates the expression and function of many proteins involved in cell growth, apoptosis, tumor suppression, and DNA repair enzymes [2, 14]. These characteristics make *tax* an essential gene in the pathogenesis of BLV. Previously, different functional areas have been described in the tax protein as a putative zinc finger motif between amino acids 30 to 53, the transactivation domain between amino acids 157 and 197, and two phosphorylation sites in residues 106 and 293 [41]; these sites are critical for modulating transcriptional activation. Amino acid substitutions in the tax protein can trigger structural and functional damage. It has been reported that C30G and A53H changes in the putative zinc finger motif region were critical and generated the loss of function as a viral transactivator [41].

The sequences of the strains in the current study were conserved between amino acids 30 to 53, except for the OP328418 Belmira strain that showed the R43K change, similar to what Ohnuki et al. [25] found. Although the R43K and A185T changes of genotype 3 are found in the putative zinc finger and leucine-rich activation domains, respectively, no data demonstrate to date the functional significance of these changes [36]. Both residues (K and T) are hydrophilic and belong to the charged polar amino acids group; glutamic acid is negatively charged, and lysine is positively charged. This may influence protein structure and its capacity as a viral transactivator. Two substitutions were found in the transactivation domain (L177V and A185T) of the OP328418 strain. The substitution of L177V may not represent damage to the structure and function of the tax protein because both residues are an apolar aliphatic chain and have a similar structure. However, substituting the alanine residue (apolar aliphatic chain) with threonine (polar without charge) can influence this domain. The region between residues 240 and 265 of the tax protein is directly related to the transcriptional activity of the virus. Its functions include regulating BLV-LTR activation, expression, in vitro but not in vivo propagation of BLV, in vitro but not alive induction of apoptosis, and restrictions on the potential of other retroviral enhancers [26, 36, 38]. Through two phylogenetic reconstruction methodologies, genotypes 1 and 3 were identified in specialized dairy herds in Antioquia, the first report of genotype 3 in Colombian dairy cows. Samples obtained from infected Holstein Friesian cows without clinically visible symptoms show the genetic variability of BLV within close geographic regions associated with nucleotide changes of the virus. Identifying new mutations in the *tax* gene (L105F, V146A, and L168V) indicates the evolutionary changes of the virus. Currently, there is no relationship between genotypes and the virulence or pathogenicity of BLV. However, these results suggest the need to conduct new analyses to establish the effect of amino acid changes on the structure and function of the envelope protein and, especially, on the tax oncogene.

## Data Availability

All data analyzed during the current study are available from the corresponding author upon request.
